# Mesenchymal Stem Cells as Therapeutic Agents and Novel Carriers for the Delivery of Candidate Genes in Acute Kidney Injury

**DOI:** 10.1155/2020/8875554

**Published:** 2020-12-11

**Authors:** Yuxiang Liu, Jingai Fang

**Affiliations:** ^1^Shanxi Medical University, No. 56, Xinjiannan Road, Taiyuan, 030001 Shanxi, China; ^2^First Hospital of Shanxi Medical University, No. 85, Jiefangnan Road, Taiyuan, 030001 Shanxi, China

## Abstract

Acute kidney injury (AKI) is a heterogeneous syndrome characterized by a dramatic increase in serum creatinine. Mild AKI may merely be confined to kidney damage and resolve within days; however, severe AKI commonly involves extrarenal organ dysfunction and is associated with high mortality. There is no specific pharmaceutical treatment currently available that can reverse the course of this disease. Notably, mesenchymal stem cells (MSCs) show great promise for the management of AKI by targeting multiple pathophysiological pathways to facilitate tubular epithelial cell repair. It has been well established that the unique characteristics of MSCs make them ideal vectors for gene therapy. Thus, genetic modification has been attempted to achieve improved therapeutic outcomes in the management of AKI by overexpressing trophic cytokines or facilitating MSC delivery to renal tissues. The present article provides a comprehensive review of genetic modification strategies targeted at optimizing the therapeutic potential of MSCs in AKI.

## 1. Background

Acute kidney injury (AKI) is a multifactorial syndrome characterized by a sharp decline in renal function and is associated with high morbidity and mortality. AKI can be triggered by a variety of predisposing factors, including sepsis, nephrotoxic drugs, and cardiac surgery. AKI affects an estimated 2% of hospital inpatients and more than 40% of critically ill patients [1]. Notably, the occurrence of AKI is steadily increasing in both developed and undeveloped nations. Despite decades of research providing deep insight into the pathogenesis of AKI, no specific options are available that can alleviate AKI or accelerate recovery; therefore, mainstay therapies remain supportive [2]. Particularly, patients who have survived AKI are at high risk for developing chronic kidney diseases and can even suffer serious health deteriorations following hospital discharge, imposing high socioeconomical burdens, particularly in low-income countries [3].

The pathophysiological mechanisms underlying AKI are complex and enigmatic [4]. Damage to the integrity of the endothelial barrier is initiating adhesion of leucocytes and platelets to the endothelium, which may be associated with microthrombi formation and microcirculatory dysfunction. Tubular epithelial cell injury has also been demonstrated to result in a rapid decline in microcirculatory function and glomerular filtration rate (GFR). Inflammatory response is crucial in mediating AKI due to both intrarenal and systemic inflammation considerably contribute to kidney injury [5]. Furthermore, it is becoming increasingly clear that the crosstalk between the immune system and kidney also plays a vital role in the development of AKI. B lymphocytes, T lymphocytes, macrophages, and dendritic cells all may be responsible for immune dysfunction after AKI. Notably, the anti-inflammatory regulatory T cells and M2 macrophages exhibiting an intrinsic renal-protective effect by alleviating inflammation, facilitating tissue repair, and remodeling in AKI [6]. Additionally, accumulating evidence indicates that mitochondrial damage is a driving factor in the progression of AKI by promoting the excess production of reactive oxygen species [7]. Pharmacological interventions aiming at one aspect of the mechanisms underlying AKI pathophysiology have failed to show beneficial effects. Thus, a novel therapeutic approach targeting multiple pathophysiological components of AKI is critically needed.

Mesenchymal stem cell (MSC) therapy is considered to have great therapeutic promise for managing AKI and has shown beneficial therapeutic effects in a variety of animal models of AKI. Kale et al. showed that MSCs infused after renal ischemia migrated specifically to injured regions and resulted in improvements in renal function [8]. Moreover, MSC transplantation improved impairments in renal function and attenuated proximal tubular epithelial cell injury, thereby facilitating recovery from AKI induced by cisplatin in a mouse model [9]. Additionally, systemically transplanted MSCs in a glycerol-induced mouse model of AKI localized to injured renal tissue and enhanced morphological and functional recovery [10].

## 2. The General Characteristics and Identification of MSCs

MSCs were first isolated from bone marrow by Friedenstein in the 1960s [11]. Subsequently, studies have shown that MSCs can be obtained from a variety of tissues, including adipose tissue, the placenta, amniotic fluid, and the umbilical cord blood [12]. For the uniform characterization of MSCs, the International Society for Cellular Therapy has developed a minimal set of criteria to define human MSCs [13]. First, MSCs should exhibit plastic-adherent characteristics when cultured in vitro. Second, MSCs must express CD90, CD73, and CD105 and cannot express CD11b, CD14, CD34, or CD45 as surface markers. Third, MSCs must possess the ability to differentiate into osteoblasts, chondrocytes, and adipocytes under appropriate induction conditions. Notably, accumulating evidence indicates that cellular surface markers and functionality are responsive to microenvironmental cues encountered in damaged tissues following MSC transplantation [14]. In addition, there are numerous researches have found that the therapeutic efficacy of MSC is primarily through paracrine mechanisms rather than cell differentiation [15].

## 3. The Mechanisms of the Action of MSCs in AKI

**Figure 1:**
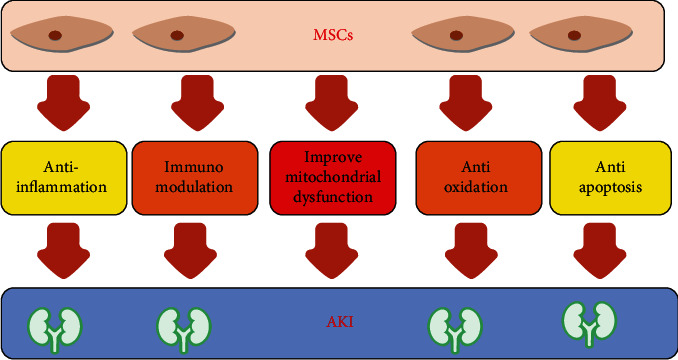
The mechanisms by which mesenchymal stem cells (MSCs) improve kidney injury.

Increasing evidence has shown that the main mechanism of the action of MSCs in AKI is the paracrine secretion of bioactive molecules by MSCs rather than MSC transdifferentiation into renal-specific cells [16]. Interestingly, Bruno et al. demonstrated that MSC-derived microvesicles (MVs) stimulated the proliferation of tubular cells and increased the resistance of tubular cells to apoptosis by transferring a specific subset of cellular mRNAs, thereby accelerating functional and morphological recovery in glycerol-induced AKI [17]. In addition, mitochondrial dysfunction is increasingly recognized as a crucial contributor to the pathophysiology of AKI [18]. A study performed by Lee et al. suggested that MSC administration prevented injury to tubular epithelial cells by reversing mitochondrial dysfunction [19]. Accumulating evidence has demonstrated that renal-immune system crosstalk plays a vital role in the development and resolution of AKI, and inappropriate immune reactions are often observed in AKI [20]. Platelet-derived growth factor identified as one of important growth factors released from MSC is crucial for cell survival and proliferation in immune cells [21]. T cells, B cells, NK cells, neutrophils, and monocytes/macrophages are pivotal in mediating injury repair and renal recovery following AKI [22]. Semedo et al. demonstrated that MSCs attenuated ischemic AKI by shifting the inflammatory response towards a Th2 profile [23]. Furthermore, MSCs mitigated AKI induced by ischemia reperfusion through interactions with splenocytes, thereby increasing the percentage of regulatory T cells in the ischemic kidney [24]. M2 macrophages are crucial in attenuating inflammatory responses as well as stimulating tissue repair after kidney injury, and MSCs can reduce rhabdomyolysis-induced AKI by skewing macrophages towards an M2 phenotype [25]. The mechanisms by which MSCs improve kidney injury are illustrated in Figure 1.

## 4. Clinical Trials with MSCs for the Treatment of AKI

In view of the promising results in different types of preclinical AKI models, several clinical trials have been performed to evaluate the safety and efficacy of MSC-based interventions for AKI patients. First, a phase 1 clinical trial was conducted to assess the safety and feasibility of allogeneic MSC administration to patients at high risk of AKI after open-heart surgery [26]. Preliminary findings showed that postoperative administration of MSCs using a dose-escalation protocol was safe, as evidenced by the absence of serious adverse events or specific adverse events related to MSC infusion. Additionally, MSC therapy reduced both the length of hospital stay and the readmission rate by up to 40%. Notably, none of the study participants treated with MSCs required renal replacement therapy, and all exhibited stable renal function, while nearly 20% of control patients developed AKI.

Moreover, Swaminathan et al. conducted a multicenter, randomized, double-blind, placebo-controlled phase 2 clinical trial to assess the safety and efficacy of allogeneic human MSC therapy for patients with AKI after cardiac surgery [27]. A total of 156 patients with AKI following cardiac surgery were enrolled in the study; 67 patients were assigned to receive intra-aortic MSCs, and 68 patients were assigned to receive the placebo. The findings revealed that no patients discontinued MSCs or placebo treatment due to serious adverse events during the study period. However, the researchers did not find significant differences between the two groups regarding the median time to recovery of kidney function, 30-day all-cause mortality, or dialysis. According to the recommendation made by the Data Monitoring Committee (DMC), the investigators stopped this clinical trial earlier than anticipated due to futility.

AKI is a multifactorial disease with high heterogeneity, which may mask the therapeutic effects of MSCs in certain subgroups of AKI patients. Therefore, additional large clinical trials in different types of AKI patients are urgently needed to shed light on the role of MSC therapy in the context of AKI. Several ongoing trials have been conducted to resolve these issues, and all registered trials on ClinicalTrials.gov regarding MSC therapy for patients with AKI are listed in Table 1.

## 5. Genetic Modification Strategies to Improve the Therapeutic Potential of MSCs

The latest findings add to a growing body of evidence that the paracrine, homing, immunomodulatory, anti-inflammatory, and tissue repair properties of MSCs can be strengthened through genetic modification [28]. It has been well established that the unique characteristics of MSCs make them ideal vectors for gene therapy [29, 30]. Thus, there has been substantially increased interest in MSC-based genetic modification strategies in recent years. In recent decades, a variety of viral and nonviral gene delivery methods have been extensively studied and widely applied for the genetic engineering of MSCs.

## 6. Viral Gene Delivery Methods

Viral transduction is one of the most commonly used methods to deliver candidate genes to MSCs [31]. Viral vectors usually include lentiviruses, adenoviruses, and adeno-associated viruses (AVVs). Lentiviral-mediated gene delivery is a highly reproducible method with high transduction efficiency that results in long-term gene expression in MSCs. Traditionally, adenoviral vector application is limited due to its inherent disadvantages, such as the lack of integration and transient target gene expression. However, a modified adenoviral-mediated gene transduction strategy has been developed that can circumvent these disadvantages [32]. Moreover, AAVs are one of the leading viral-mediated gene delivery methods that can be used to import DNA sequences of interest into MSCs [33]. Although viral-mediated gene delivery techniques allow relatively stable transgene expression and high efficiency, their utilization remains challenging due to a variety of concerns, such as carcinogenicity, immunogenicity, toxicity, and high costs [34]. By performing a more detailed assessment of immune reactions to various viral vectors, intensive research to overcome the limitations associated with viral-mediated gene delivery methods will build a foundation for future clinical practices.

## 7. Nonviral Gene Delivery Methods

Nonviral gene transfer techniques commonly consist of physical and chemical methods [35]. Physical methods include microinjection, electroporation, and sonoporation. Microinjection is a relatively safe, inexpensive, and nontoxic method, but its application is limited because large micropipettes may result in marked cell damage. Electroporation is a highly reproducible method with the ability to transfer large genes, and the major concern with this technique is that the high voltage utilized in electroporation may contribute to cell damage [36]. Sonoporation is a promising gene delivery technique; however, MSCs transfected by sonoporation exhibit low gene transfer efficiency [37]. In addition to physical methods, a variety of chemical methods have been developed and used to deliver nucleic acid materials containing siRNA, mRNA, and DNA into MSCs via cationic lipids, cationic polymers, and inorganic nanoparticles [38]. Compared to those of viral transduction methods, the major disadvantages associated with nonviral gene delivery methods are transient transgene expression and relatively low gene transfer efficiency.

## 8. Application of genetically modified MSCs in preclinical AKI models

To achieve improved therapeutic outcomes in the management of AKI, genetic modification has been attempted to overexpress trophic cytokines or facilitate MSC delivery to renal tissues. A variety of target genes have been engineered for transfection into MSCs and applied in preclinical AKI models. These tested genes are summarized in Table 2.

## 9. CXC-Chemokine Receptor Type 4

CXC-chemokine receptor type 4 (CXCR4) is the receptor for stromal cell-derived factor-1 (SDF-1), and the SDF-1/CXCR4 axis has been shown to govern the migration of MSCs towards injured tissue in a variety of species and tissue types [39]. SDF-1 was shown to be significantly upregulated in response to ischemic conditions, including AKI. However, expression of CXCR4 was observed only in a small fraction of MSCs, and CXCR4 expression was significantly decreased during ex vivo expansion, which may result in the poor homing efficiency of transplanted MSCs to injured kidney tissues, thereby limiting their therapeutic potential. A variety of studies have focused on overexpressing CXCR4 to facilitate MSC mobilization [40]. Target overexpression of CXCR4 in MSCs was shown to significantly enhance the migration of MSCs to the injured kidney, as well as markedly reduce acute tubular necrosis scores and exert beneficial effects on renal function [41].

## 10. Erythropoietin

Erythropoietin, a hemopoietic growth factor secreted mainly by the kidney, is a pleiotropic cytokine with tissue-protective and reparative properties. Mounting evidence shows that erythropoietin is a vital modulator that is involved in a variety of biological processes, including cellular mitogenesis, maturation, survival, DNA repair, and angiogenesis [42]. In addition, erythropoietin is recognized to be a renotropic factor that supports tubular repair and accelerates renal functional recovery through erythropoietin receptors in the context of kidney injury [43]. The administration of exogenous erythropoietin to rats at the onset of AKI induced by bilateral renal artery occlusion exerted renoprotective effects by alleviating apoptosis, ameliorating renal dysfunction, and increasing tubular regenerative capacity [44]. Notably, MSCs overexpressing erythropoietin via retroviral transduction promoted renal epithelial cell survival, as measured by flow cytometry to assess annexin V and propidium iodide (PI) expression after cisplatin exposure in vitro [45]. Moreover, Epo-MSCs showed additional favorable effects on kidney function and survival in a mouse model of cisplatin-induced AKI [45].

## 11. Kallikrein

Tissue kallikrein, a critical member of the kinin-releasing enzyme family, is a serine protease expressed in many cell types. Basic research has demonstrated that the kallikrein-kinin system (KKS) is implicated in the physiopathological mechanisms of cardiovascular disease and the inflammatory response [46]. Notably, numerous studies have shown that kallikrein exerts nephroprotective effects in a range of disease models, including models of diabetes, postischemic heart failure, and acute cardiac ischemia [47]. Adenoviruses harboring the human kallikrein gene, when used for gene transfer, significantly decreased oxidative stress and TGF-*β* expression, thereby attenuating salt-induced renal fibrosis, as identified by reduced collagen deposition and renal fibrosis scores [48]. Based on the above research, kallikrein may be a therapeutic target for ameliorating kidney injury. Intriguingly, MSCs genetically modified with the kallikrein gene were more effective in reducing inflammatory cell infiltration, decreasing reactive oxygen species formation, and mitigating ischemia-induced renal injury than unmodified MSCs due to the combination of MSC-mediated paracrine effects and the pleiotropic properties of kallikrein [49].

## 12. Vascular Endothelial Growth Factor

Vascular endothelial growth factor (VEGF) is a proangiogenic cytokine that initiates the proliferation and migration of endothelial cells, thereby contributing to the generation and stabilization of new blood vessels [50]. An integrated vascular network is critical for maintaining renal physiological functions, and studies have demonstrated that the inhibition of VEGF bioactivity is followed by podocyte injury and results in a defective renal structure [51]. VEGF is a principal paracrine cytokine that mediates renoprotective effects following AKI in the context of MSC therapy. The therapeutic potential of MSCs was decreased considerably after knockdown of VEGF via siRNA in a rat model of ischemic AKI [52, 53]. Additionally, Yuan et al. investigated the effect of VEGF-modified human embryonic MSCs on cisplatin-induced AKI in a nude mouse model [54]. In that study, cellular apoptosis, peritubular capillary density, renal function, and tubular structure were examined, and the results demonstrated that VEGF upregulation strengthened the renoprotective effect of MSCs in the context of these parameters [54].

## 13. Hepatocyte Growth Factor

Hepatocyte growth factor (HGF) is generated by cells of mesodermal origins and primarily acts on epithelial and endothelial cells to produce a wide range of biological effects, including the promotion of cellular growth, the stimulation of cellular motility, and the induction of tissue regeneration [55]. The circulating concentration of HGF is markedly increased following renal injury. Subcutaneous injection of HGF into rats markedly enhanced renal function recovery and tubular regeneration after AKI induced by bilateral renal artery occlusion [56]. Moreover, with the administration of recombinant human HGF in mice facilitated DNA synthesis in renal tubular cells and promoted remodeling of renal tissue after HgCl2-mediated renal injuries [57]. Evidence indicates that HGF acts in a renotropic manner to induce kidney regeneration after acute injury [58, 59]. Notably, Chen et al. established MSCs overexpressing HGF by using an adenoviral vector and then administered these MSCs into rats following ischemia/reperfusion- (I/R-) induced AKI [60]. Their results showed that HGF-MSC-treated rats exhibited further improvements in renal cell regeneration, inflammatory responses, and renal functions compared with those of MSC-treated rats [60].

## 14. Nuclear Factor E2-Related Factor 2

Nuclear factor E2-related factor 2 (Nrf2) is an important transcription factor that activates a range of antioxidant responsive element- (ARE-) driven genes, thereby contributing to the modulation of the cellular antioxidant defense system [61]. In recent decades, abundant evidence has suggested that the oxidative stress response plays a critical role in the injury phase of AKI. However, the poor survival of transplanted MSCs in the oxidative stress microenvironment of renal tissues is the principal obstacle faced by the current MSC management strategy for AKI [62]. Interestingly, Nrf2(+/+) bone marrow stromal cells exhibited increased resistance to oxidative and electrophilic stress compared to Nrf2(-/-) stromal cells [63]. Nrf2-overexpressing MSCs were established by using recombinant adenoviruses and transplanted into rats with AKI induced by cisplatin, and the results demonstrated that the transplantation of Nrf2-MSCs resulted in a superior therapeutic effect on the complications of AKI [64]. Furthermore, Zhaleh et al. demonstrated that plasmid-mediated overexpression of Nrf2 in MSCs downregulated the levels of injury markers and upregulated the levels of repair-induced genes after transplantation in rats suffering from glycerol-induced AKI [65].

## 15. Klotho

Klotho is a membrane-bound protein that was originally identified as a suppressor of aging-related genes. Klotho is emerging as both a pharmaceutical agent and a highly promising biomarker for AKI [66]. Prior administration of adenoviruses harboring the Klotho gene significantly attenuated apoptosis in mice subjected to bilateral renal ischemia [67]. In addition, Klotho is a crucial suppressor of renal fibrosis, and its antifibrotic effect is mediated by the inhibition of Wnt signaling [68]. The available evidence supports the hypothesis that targeted renal delivery of Klotho via MSC transplantation could have a strong reparative effect on AKI. Zhang et al. demonstrated in a mouse model that the administration of Klotho gene-modified bone marrow-derived MSCs more significantly mitigated kidney fibrosis resulting from ischemia/reperfusion injury (IRI) than that of unmodified MSC transplantation [69]. Additionally, the protective effects of bone marrow-derived MSCs on renal IRI were considerably increased by overexpressing the Klotho gene, as determined by TUNEL staining and serum creatinine assessment [70].

## 16. Heme Oxygenase-1

Heme oxygenase-1 (HO-1) is a rate-limiting enzyme that participates in heme breakdown, leading to the production of iron, biliverdin, and carbon monoxide. These degradation byproducts regulate a wide array of vital biological functions that involve anti-inflammation, antioxidation, and cellular protection [71]. Accumulating evidence suggests that HO-1 plays a critical cytoprotective role in different types of kidney disease. Of note, experimental studies have demonstrated that a variety of therapeutic agents for the treatment of AKI exert their protective effects by inducing the generation of HO-1 [72]. However, HO-1-deficient mice were more susceptible to renal function deterioration and tubular injury after cisplatin-induced AKI [73]. HO-1 overexpression by a lentiviral vector greatly improved the survival of MSCs in the context of AKI induced by I/R, which was accompanied by significant improvements in renal function and an improved anti-inflammatory effect [74]. These authors also reported that HO-1-overexpressing MSCs exhibited increased proliferation and improved differentiation abilities in the AKI microenvironment [75].

## 17. Other Strategies to Enhance the Therapeutic Efficacy of MSCs

In addition to genetic manipulation, another strategy which is aimed at enhancing the therapeutic potential of MSCs is to precondition MSCs in vitro. In particular, culturing MSCs under hypoxic conditions in vitro can enhance the secretion of trophic factors and their migratory capacity [76]. Yu et al. demonstrated that hypoxic preconditioning of MSCs resulted in greater improvements in renal function in a rat model of ischemic AKI by alleviating apoptosis and promoting angiogenesis [77]. The efficacy of MSCs is critically affected by their source of origin, number of passages, and delivery route. Adipose-derived MSCs perform better than bone marrow-derived MSCs in terms of stimulating the production of anti-inflammatory factor interleukin-10 (IL-10) by dendritic cells [78], which may bring incremental benefits to AKI. Moreover, pluripotent stem cell-(PSC-) derived MSCs possess better of cell quality with batch-to-batch consistency and higher proliferative potential, exhibiting superior therapeutic benefits for tissue regeneration to BM-derived MSCs [79]. Recently, induced pluripotent stem cell- (iPSC-) derived MSCs have been applied in steroid-resistant acute graft versus host disease and demonstrated good safety and tolerability in clinical trials [80]. iPSC-derived MSCs may provide another putative cellular source overcome many limitations of adult MSC used in immune disorder-provoked AK. There are many disadvantages of using adult MSC (i.e., BM-MSC) including stem cell senescence. Liang et al. found that rejuvenation of aged MSC via overexpressing Erb-B2 receptor tyrosine kinase 4 (ERBB4) could enhance angiogenesis via PI3K/AKT and MAPK/ERK pathways [81], which may improve the potential efficacy of MSCs in treating AKI. Bustos et al. demonstrated that aged MSCs lacked the anti-inflammatory protective effect compared to their young counterparts, implying young MSCs may display a superior protective role in diseases [82]. The route of MSC administration is commonly systemic transplantation; while only a small proportion of MSCs migrated to injured renal tissue after infusion, most of the cells were trapped in other organs such as lung, spleen, and heart. It is reasonable to assume that local injection may be another approach to enhance the therapeutic efficacy of MSCs.

## 18. Concerns regarding Genetically Modified MSC Therapy in AKI

It would take quite a long time to harvest enough gene-modified MSCs due to the relatively complex genetic transfection procedure. Notably, the study performed by Lee et al. showed that the levels of stress- and apoptosis-related genes in MSCs were markedly increased over time [83]. Thus, time is a vital element to be considered when transplanting genetically modified MSCs because older MSCs exhibit weakened protective effects [82]. Little is currently known about the long-term behavior of genetically modified MSCs in vivo and the long-term impacts of genetic manipulation on the characteristics of MSCs, such as the self-renewal, differentiation, and paracrine properties. Unfortunately, viral vectors and their transgene products may induce innate and adaptive immune responses that limit or even offset the therapeutic potential of gene therapy [84, 85]. Systematic inflammatory responses triggered by adenoviral vectors negatively affect long-term gene expression and genetic stability [86]. Ideally, MSCs should be administered quickly at the onset of AKI. However, the initial phase of AKI lacks typical symptoms and established biomarkers, making it difficult to apply MSCs as soon as possible. Additionally, AKI is a heterogeneous condition, and so identifying a subgroup of patients who are responsive to MSC-based gene therapy may be extremely crucial in producing the desired efficacy.

## 19. Conclusions

Genetic modification of MSCs represents a promising and effective approach to enhance the therapeutic potential of these cells for AKI treatment. However, among the multiple target genes listed in this review, it remains unknown which gene, after modification in MSCs, will achieve an ideal outcome for the management of AKI. With a more comprehensive understanding of the potential drawbacks and obstacles of genetically modified MSC therapy for AKI, additional efforts will be needed to address these issues prior to the use of MSC-based gene therapy to produce results that can be translated into clinical practice.

## Figures and Tables

**Table 1:** Registered clinical trials involving the use of MSCs in AKI.

ClinicalTrials.gov Identifier	Study title	Study phase	Actual/estimated enrollment	MSC source	Primary outcome	Location	Status
NCT04194671	Clinical Trial of Mesenchymal Stem Cells in the Treatment of Severe Acute Kidney Injury	Phase 1/2	80 participants	Umbilical cord	Progression of renal function within 28 days after receiving MSC	China	Not yet recruiting
NCT01275612	Mesenchymal Stem Cells in Cisplatin-Induced Acute Renal Failure in Patients with Solid Organ Cancers	Phase 1	0 participants	Bone marrow	The rate of renal function loss up to 15 days postcisplatin infusion	Italy	Withdrawn
NCT03015623	A Study of Cell Therapy for Subjects with Acute Kidney Injury Who are Receiving Continuous Renal Replacement Therapy	Phase 1/2	24 participants	Unclear	Outcomes out to day 28 and serious adverse events through day 180	United States	Recruiting
NCT01602328	A Study to Evaluate the Safety and Efficacy of AC607 for the Treatment of Kidney Injury in Cardiac Surgery Subjects (ACT-AKI)	Phase 2	156 participants	Bone marrow	Time to kidney recovery	United States	Terminated
NCT00733876	Allogeneic Multipotent Stromal Cell Treatment for Acute Kidney Injury following Cardiac Surgery	Phase 1	15 participants	Unclear	MSC-specific adverse or serious adverse events	United States	Completed
NCT02563366	Effect of BM-MSCs on Early Graft Function Recovery after DCD Kidney Transplant	Phase 1/2	120 participants	Bone marrow	Estimated glomerular filtration rate at one month posttransplant	China	Unknown
NCT02561767	Effect of BM-MSCs in DCD Kidney Transplantation	Phase 1/2	120 participants	Bone marrow	Estimated glomerular filtration rate at one month posttransplant	China	Unknown

**Table 2:** Research regarding the application of gene-modified MSCs in preclinical AKI models.

Candidate gene	AKI model	Vector	MSC source	Effects	Reference
CXCR4	Mice model of I/R AKI	Lentiviral vector	Bone marrow	(i) Promote MSC paracrine actions in vitro (ii) Accelerate migratory of MSCs to damaged renal tissue (ii) Improves treatment with BMSCs in I/R-AKI mice	[41]
EPO	Mouse model of cisplatin-induced AKI	Retroviral vector	Bone marrow	(i) Improve survival of mouse kidney epithelial cells in vitro (ii) Enhance antiapoptotic and anti-inflammatory effects (iii) Improve survival of cisplatin-treated mice	[45]
Kallikrein	Rat model of I/R AKI	Adenoviral vector	Bone marrow	(i) Reduce inflammatory cell infiltration (ii) Alleviate reactive oxygen species formation (iii) Mitigate ischemia-induced renal injury	[49]
VEGF	Mice model of cisplatin-induced AKI	Adenoviral vector	Embryo	(i) Reduce renal cell apoptosis (ii) Increase renal microvessel density (iii) Reduce mortality of AKI mice	[54]
HGF	Rat model of I/R AKI	Adenoviral vector	Umbilical cords	(i) Promote the amelioration of renal function (ii) Improve the proliferation of renal cells (iii) Decrease apoptosis and inflammation	[60]
Nrf2	Rat model of cisplatin-induced AKI	Adenoviral vector	Bone marrow	(i) Protect MSCs against cisplatin-induced toxicities in vitro (ii) Improve efficacy of MSCs against cisplatin-induced toxicity	[64]
Klotho	Mice model of I/R AKI	Adenoviral vector	Bone marrow	(i) Improve antifibrotic effect in kidney (ii) Mitigate the degree of renal histopathological injury (iii) Inhibit epithelial-mesenchymal transition	[69]
HO-1	Rat model of I/R AKI	Lentiviral vector	Bone marrow	(i) Promote anti-inflammatory effect of MSCs in vitro (ii) Protects BMSCs against apoptosis in vivo	[74]
